# Complete Genome Sequence of Streptococcus thermophilus VHProbi R08, Isolated from Fermented Sour Porridge

**DOI:** 10.1128/mra.01196-22

**Published:** 2023-01-04

**Authors:** Kailing Li, Hongchang Cui, Zhi Duan

**Affiliations:** a Qingdao Vland Biotech Group Co., Ltd., Qingdao, China; University of Rochester School of Medicine and Dentistry

## Abstract

Streptococcus thermophilus VHProbi R08 is a bacterial strain isolated from fermented sour porridge in northern China. Here, we report the complete genome sequence of VHProbi R08, which comprises 1,848,461 bp, 1,906 protein-coding genes, 57 tRNA genes, and 15 rRNA genes.

## ANNOUNCEMENT

The species Streptococcus thermophilus is a kind of starter culture for the dairy industry (1) and is one of the predominant lactic acid bacteria, with rapid acidification capability (2, 3). Fermented sour porridge (1 g) was serially diluted in NaCl solutions (0.85%) and spread onto modified Chalmers agar (Luqiao, China). The inoculum was incubated under aerobic conditions at 37°C for 48 h. One red colony was isolated and identified as the species S. thermophilus by 16S Sanger sequencing (4).

A combined sequencing strategy using the Illumina HiSeq 2500 and PacBio RS II platforms was used to sequence the whole genome of S. thermophilus VHProbi R08. The strain was inoculated into M17 medium (Luqiao, China) and cultured at 37°C for 24 h to extract DNA. Total DNA was extracted using a genomic DNA purification kit (Promega, USA) following the manufacturer’s instructions. Template DNA was sheared into 400-bp fragments for Illumina library creation using the NEXTflex rapid DNA-seq kit (Bioo Scientific, USA). Then, the prepared library was used for paired-end Illumina sequencing (2 × 150 bp). For PacBio sequencing, an aliquot of 15 μg DNA was spun in a g-TUBE (Covaris, MA) to obtain fragments. DNA fragments were then purified, end repaired, and ligated with SMRTbell (PacBio, CA) sequencing adapters, following the manufacturer’s recommendations. Next, an ~10-kb insert library was prepared and sequenced on one single-molecule real-time (SMRT) cell using standard methods. Finally, 7,249,570 raw reads and 7,107,752 clean reads were generated. Using the PacBio RS II sequencing platform, 82,164 raw reads (*N*_50_, 7,780 bp) were generated. The Illumina raw reads were quality filtered using Sickle v1.33 (https://github.com/najoshi/sickle), and the low-quality reads were removed before assembly. The Illumina raw reads were assembled into a draft genome sequence using SOAPdenovo (5). The PacBio raw reads were then assembled into a complete genome using Unicycler v0.4.8 (6). Pilon was used for error checking the PacBio assembly results and judging the start site of the circular chromosome using the Illumina assembly result (7). The last circular step was checked manually using Pilon: there was an overlap of 5,000 bp at both ends of the contig, so one end of the overlap was cut off to loop the sequence. Glimmer v3.02 (8), tRNAscan-SE v2.0 (9), and Barrnap v0.9 were used to predict the protein-coding genes, tRNAs, and rRNAs, respectively (https://github.com/tseemann/barrnap/). CheckM v1.1.3 was used to check the quality of the assembled genome sequence, yielding a completeness of 99.89%, contamination of 0.15%, and strain heterogeneity of 0% (10). Genome annotation was conducted using the Prokaryotic Genome Annotation Pipeline (PGAP) v5.3 (11). Default parameters were used for all software unless otherwise specified.

The complete genome sequence of this strain contains a chromosome with 1,848,461 bp. The depth of coverage of the final assembled genome was 342.3-fold. The chromosome has a CG content of 39.06%. Furthermore, it contains 1,906 protein-coding genes, 57 tRNA genes, and 15 rRNA genes. The genome sequence indicates that this strain contains an 18.9-kb bacteriocin gene cluster that includes 22 genes.

### Data availability.

The complete genome sequence of S. thermophilus VHProbi R08 has been deposited at GenBank under accession number CP094945, SRA accession numbers SRR18904151 and SRR18904150, BioProject accession number PRJNA821985, and BioSample accession number SAMN27162337.
